# Different Mechanisms for Heterogeneity in Leprosy Susceptibility Can Explain Disease Clustering within Households

**DOI:** 10.1371/journal.pone.0014061

**Published:** 2010-11-19

**Authors:** Egil Fischer, Sake De Vlas, Abraham Meima, Dik Habbema, Jan Richardus

**Affiliations:** Department of Public Health, Erasmus MC, University Medical Center Rotterdam, Rotterdam, The Netherlands; Direccion General de Epidemiologia, Peru

## Abstract

The epidemiology of leprosy is characterized by heterogeneity in susceptibility and clustering of disease within households. We aim to assess the extent to which different mechanisms for heterogeneity in leprosy susceptibility can explain household clustering as observed in a large study among contacts of leprosy patients.

We used a microsimulation model, parameterizing it with data from over 20,000 contacts of leprosy patients in Bangladesh. We simulated six mechanisms producing heterogeneity in susceptibility: (1) susceptibility was allocated at random to persons (i.e. no additional mechanism), (2) a household factor, (3, 4) a genetic factor (dominant or recessive), or (5, 6) half a household factor and half genetic. We further assumed that a fraction of 5%, 10%, and 20% of the population was susceptible, leading to a total of 18 scenarios to be fitted to the data. We obtained an acceptable fit for each of the six mechanisms, thereby excluding none of the possible underlying mechanisms for heterogeneity of susceptibility to leprosy. However, the distribution of leprosy among contacts did differ between mechanisms, and predicted trends in the declining leprosy case detection were dependent on the assumed mechanism, with genetic-based susceptibility showing the slowest decline. Clustering of leprosy within households is partially caused by an increased transmission within households independent of the leprosy susceptibility mechanism. Even a large and detailed data set on contacts of leprosy patients could not unequivocally reveal the mechanism most likely responsible for heterogeneity in leprosy susceptibility.

## Introduction

Leprosy, caused by infection with *Mycobacterium leprae*, was detected in a quarter of a million people in 2008, and many more people are living with impairments caused by this disease.[1] Although the WHO goal of an on-treatment prevalence of less than 1 per 10,000 was reached world-wide[2], in many countries or regions case detection rates are well above this goal.[1]

Clustering of leprosy patients within households, families, and neighborhoods has been reported many times.[3], [4], [5], [6], [7], [8] This clustering is partly due to a higher contact intensity, hence an elevated possibility of transmission between contacts. However, only a few people that are exposed to the infection, within or outside households, actually develop the disease.[9], [10], [11] Introduction of leprosy on an island shows the heterogeneity in susceptibility most clearly, as the number of cases is limited to a proportion of the total population, smaller than expected given the initial rapid increase of cases.[12] The fraction of susceptible members of the population of the Indian subcontinent is thought to be approximately 10%.[9], [10], [13] We hypothesize that the clustering of leprosy in households is due to a combination of the increased exposure to infection and specific mechanisms that cluster susceptibility within households.

Association between genetic elements and leprosy susceptibility has been suggested previously.[14], [15] Leprosy develops in a spectrum of clinical forms, from self-healing to the chronic lepromatous type of leprosy. Recent studies with whole-genome screening in Viet Nam and Brazil indicate a two-step genetic mechanism in which leprosy susceptibility and the type of leprosy are determined by alleles of genes on different chromosomes.[16], [17] Epidemiological studies support the existence of a genetic factor for the risk of leprosy[5], [7], but these studies are not conclusive due to the fact that familial relationship and household membership are correlated.[5]

Another mechanism determining heterogeneity in leprosy susceptibility may explain the observed clustering of leprosy in household contacts. In Moet *et al.* [7], the odds-ratio of having leprosy for close relatives is only marginally significant after adjusting for contact distance, *e.g.* household member, neighbor, or social contact. This result indicates that the risk of family members might be caused by a common (but yet unknown) risk factor in a household, such as poverty, which in Brazil has been shown to be a risk factor for leprosy.[18]

In this study, we analyze the data from the study by Moet *et al.* [7] to quantify the level of within- and between-household transmission of *M. leprae*. Using a newly-developed microsimulation model, we attempt to distinguish between mechanisms causing heterogeneity in leprosy susceptibility. The study of Moet *et al.* [7] was performed in the context of a randomized controlled trial of the effect of chemoprophylaxis, and contains the data of 21,870 contacts of 1,037 leprosy patients detected by a rural health program in northwest Bangladesh. In this region, the new case detection rate has been declining in the last decades. This large and detailed dataset, in combination with our model, is used to investigate six mechanisms for heterogeneity of leprosy susceptibility. We assess the extent to which the distribution of cases among households can be explained by different mechanisms, with the ultimate goal of identifying the most likely ones.

## Methods

### Modeling leprosy and a household-structured population

We used microsimulation modeling, a technique in which life histories of fictitious individuals are simulated. Individual humans are the unit of modeling, and dynamics at the population level are obtained by aggregation of all individuals. Microsimulation has been employed for studies of infectious diseases with complex natural histories or complex patterns of individual contacts, *e.g.* helminthic parasites[19], sexually transmitted diseases[20], [21], malaria[22], influenza[23], and bovine tuberculosis[24]. To reduce the computation time of our microsimulation model, we made use of a recently developed method which increases the variation of the model outcomes, but gives a good approximation of the average outcome.[25]

Our model, called SIMCOLEP, simulates the spread of *M. leprae* in a population divided into households, and the development of leprosy by infected individuals. The model is based on and parameterized for the population[26] and leprosy epidemiology[7] in Nilphamari and Rangpur, Bangladesh. The data of leprosy amongst household contacts was derived from a large trial on chemoprophylactic treatment of contacts[7].This trial included over 20,000 contacts of over 1,000 newly detected patients in the period June 2002-December 2003. For a full description of SIM*CO*LEP and details of parameterization, see the Supporting Information File S1.

Demography is described by birth, death, and movement between households. A life table determines the life span of an individual. At birth, individuals are placed in the household of their mother, and individuals can move from one household to another existing or newly-created household during their lifetime. Individuals move at marriage, or during adolescence.[26]

Transmission occurs due to direct contact with infectious individuals. An infectious individual makes infectious contact with random individuals in the population at rate *c_pop_* (contacts per year), multiplied by the probability of infection during a contact, *i.e.* the infectivity. Additionally, within a household containing one or more infectious individuals, each susceptible household member is infected at the minimum of times until an infectious contact from each infectious individual in the household. The timing of these infectious contacts is determined by the rate *c_hh_* (contacts per year), and the infectivity. These two contact rates, *c_pop_* and *c_hh_*, are estimated by fitting the model to the data.

**Figure 1:**
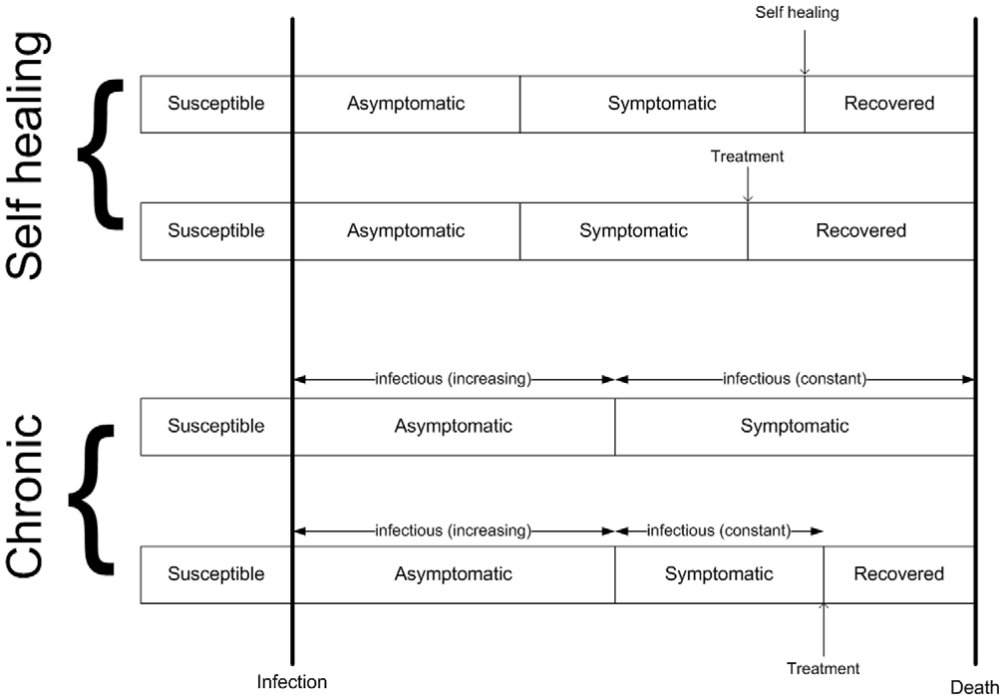
Natural history of infection from birth until death, for self-healing and chronic leprosy in the model. Both types of leprosy, self-healing and chronic, start in a susceptible state. Self-healing enters an asymptomatic state, progresses to the symptomatic or clinical state, is followed by self-healing or treatment, and finally transitions to the recovered state. The chronic form enters a different asymptomatic state after infection. Here, the infectivity, *i.e.* the probability of transmission during an adequate contact, increases with the duration in this state. When progressing to the symptomatic state, the infectivity reaches the maximum and remains constant. The individual will stay in this symptomatic state until death unless treatment is provided. Treatment results in a transition to the recovered state, in which the individual is no longer infectious.

The natural history of the infection, schematically shown in Figure 1, is modeled following the model of Meima *et al*.[13]. Only susceptible individuals can become infected. We model two types of leprosy: either self-healing or chronic. After acquiring infection, the individual enters the asymptomatic state. Chronic infection will later progress to the symptomatic state, remaining until the individual dies or is treated. The chronic infection is infectious during both the asymptomatic and symptomatic states, with infectivity increasing linearly during the asymptomatic state. Together with the contact rates, *c_pop_* and *c_hh_*, the infectivity determines the rate at which new infectious contacts are made. Self-healing infections are never infectious, and proceed to the recovered state at the end of the symptomatic period. Both chronic and self-healing leprosy can be detected while symptomatic, subsequently treated, and cured.

We mimic the leprosy situation in the Nilphamari and Rangpur districts and thus also the control programs. Treatment becomes available in 1970, after which the average detection delay decreases from 12 years to 2 years in 1990.[13] Treatment in 1970 starts with dapsone monotherapy and is gradually replaced by multi-drug therapy (MDT) since 1985. MDT is fully implemented by 1990. The relapse rate decreases from 0.015 to 0.001 per year.[27], [28] From 1990 onwards, household members of newly detected patients are examined. Vaccination with Bacillus Calmette-Guérin (BCG) is protective against leprosy with a protective effect of 60%.[29], [30] BCG vaccination begins in 1974 with an initial coverage of 40%, rising to 80% in 1990[31].

### Scenarios for heterogeneity of susceptibility in the population

We model a population in which a small fraction (5%, 10%, or 20%) is susceptible to leprosy. The majority of the population is not susceptible; these individuals do not develop symptoms and are never infectious. Allocation of susceptibility and the type of leprosy (self-healing or chronic) follows one of six mechanisms, which will be explained in more detail below, and is summarized in Table 1. In total, 18 scenarios – *i.e.* six mechanisms multiplied by three fractions of susceptibles – are fitted to data.

**Table 1:** Description of the six mechanisms determining the heterogeneity in susceptibility.

Mechanism	Description
*Random*	Equal probability for each individual, i.e. random allocation of susceptibility
*Household*	Random sample of individuals in randomly selected households (25% of all households)
*Dominant*	A dominant gene inherited from one or both parents
*Recessive*	A recessive gene inherited from both parents
*Household & dominant*	50:50 distribution of susceptibility by:1. A dominant gene inherited from one or both parents or 2. A random sample of individuals in 25% randomly selected households
*Household & recessive*	50:50 distribution of susceptibility by: 1. A recessive gene inherited from both parents or 2. A random sample of individuals in 25% randomly selected households

The simplest mechanism causing heterogeneity in leprosy susceptibility is random distribution of susceptibility over the population. We will indicate this mechanism with “*Random*”. In the second mechanism, indicated by “*Household*”, the inhabitants can be susceptible in 25% of the households[32] due to a common factor within their shared household, such as poverty. Not all members of a susceptible household are susceptible, allowing for variation within households. The fraction of susceptibles within a household, multiplied by the 25% of households yields the total fraction of susceptibles in the population. For both the *Random* and the *Household* mechanisms, 80% of susceptibles display self-healing leprosy (determined by chance), and the remaining 20% develop the chronic type. The third and fourth mechanisms are genetic: Mendelian inheritance of one gene determining leprosy susceptibility, and a second gene determining leprosy type (self-healing or chronic). We consider two mechanisms where either both genes are dominant (“*Dominant*”), or both genes are recessive (“*Recessive*”). Finally we considered two mechanisms in which a household factor is combined with a dominant or a recessive genetic factor (“*Household* & *dominant*” and “*Household* & *recessive*”). Half of leprosy susceptibility is caused by a genetic mechanism, and the other half is due to living in a susceptible household.[5]

### Fitting the model to data

The data used for fitting the model are the new case detection rate (number of new cases per 10,000), the prevalence of cases among contacts for different household sizes, and the prevalence of cases among different classes of relatives.[7] For each combination of the *c_pop_* and *c_hh_* contact rates within an 11 by 11 parameter grid, we calculate the log-likelihood of the outcomes of 100 simulation runs for the leprosy data in 2003.[7] To determine the parameter values with the highest likelihood, a regression model was fitted to the outcomes of the simulated grid points.[33] New simulations were performed with the most-likely parameter values to determine the detailed outcomes of the model at those parameter values. For each mechanism, simulations were continued until the year 2020 to predict future trends in new case detection. The Supporting Information File S1 includes a detailed description of the fitting procedure, and outcomes of the simulations.

## Results

**Figure 2:**
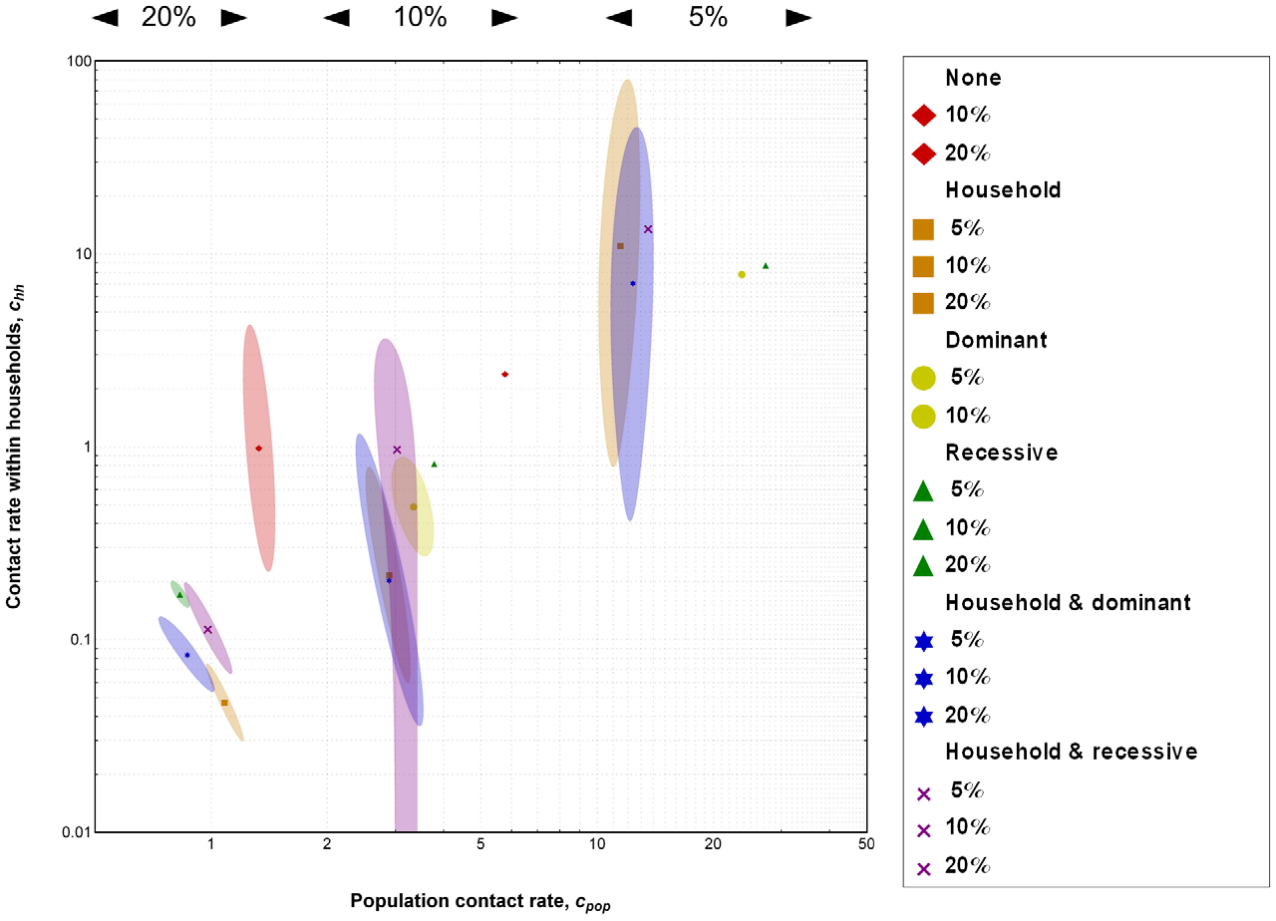
Best-fitting parameter combinations for the rate at which infectious contact is made in the population (contacts per year), *c_pop_*, and the contact rate within a household (contact per year), *c_hh_*, for six mechanisms of heterogeneity in leprosy susceptibility and three fractions of susceptibles. The within-household transmission is transmission on top of the population level transmission. The markers indicate the best fit for each scenario. The shaded areas in the same color indicate the area in which the fit did not differ from the overall best fitting scenario (*P*>0.01); not all mechanisms had an area with *P*>0.01. The mechanisms *Random* (5% susceptibles) and *Dominant* (20% susceptibles) could not be fitted to the data, and are thus not shown.

Each of the six mechanisms could be fitted to the data for one or more of the fractions of susceptibles in the population (Figure 2). The assumed fraction of susceptibles in the population (5%, 10%, or 20%) determines, to a large extent, the value of the population contact rate, *c_pop_*. This rate plays the predominant role in fitting the new case detection data (data not shown). Two scenarios, the *Random* mechanism with 5% susceptibles in the population, and *Dominant* with 20% of the population susceptible, could not be fitted to the data. We observed that for *Random*, the within-household transmission rate, *c_hh_*, and the contact rate in the population, *c_pop_*, are high compared to the other mechanisms in which susceptibility is clustered within households (Figure 2). This result demonstrates an amplifying effect of clustering of susceptibility. More details of the fitting including figures of the simulations can be found in the Supporting Information File S1.

**Figure 3:**
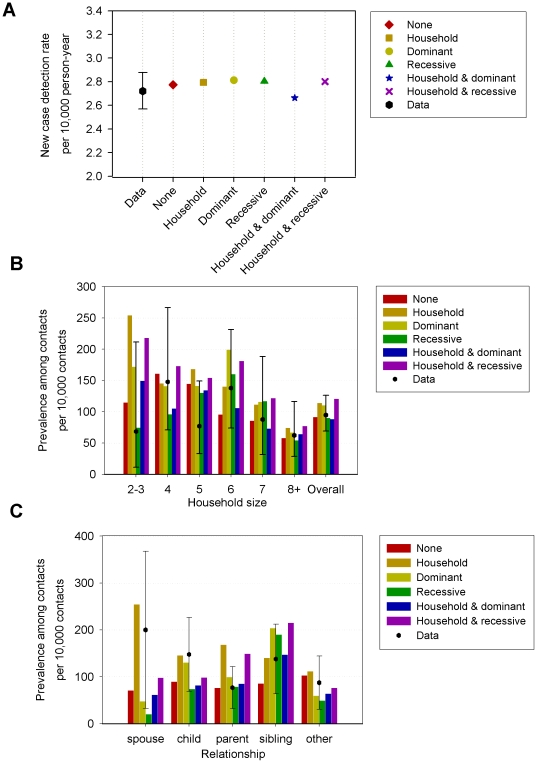
Comparison of model output with observations for six mechanisms of heterogeneity in leprosy susceptibility, assuming 10% susceptibles in the population. A value of 20% susceptibles provided a much better fit for the *Random* mechanism. (A) New case detection rate per 10,000 inhabitants. The observed detection rate in 2003 is shown on the left, with 95% confidence interval. In total 1,184 patients in a population of 4.3 million persons. (B) Prevalence of leprosy among previously undiagnosed contacts of leprosy patients by household size. (C) Prevalence of leprosy among previously undiagnosed contacts of leprosy patients by relationship to the index patient. For (B) and (C) were used 43 previously undiagnosed contacts in 1,034 households.

Even though the mechanisms provide a comparable overall fit to the data, there are substantial differences in which aspects of the data are fitted best (Figure 3). For example, the prevalence among contacts by household size is similar for all household sizes in the *Random* mechanism, while the pattern for *Household* is skewed to low household size, and the genetic mechanisms show a peak at households of size six (Figure 3B). The distribution of cases among types of relationships also displays marked differences (Figure 3C). The *Household* mechanism results in a high prevalence among spouses, while the genetic mechanisms underestimate the prevalence among spouses. The genetic mechanisms differ in the prevalence among siblings, children, and parents. The combined mechanisms are not always intermediate in comparison to those for the *Household* and genetic mechanism (*Recessive* or *Dominant)*.

**Figure 4:**
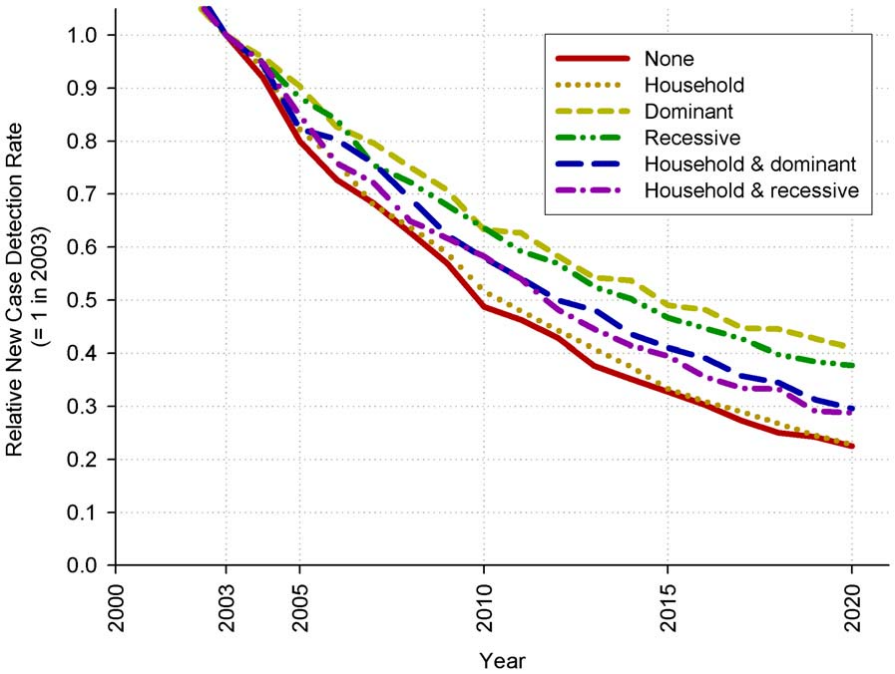
Trend in decline of the leprosy new case detection rate, relative to the new case detection rate in 2003 (value is 1 in 2003). The assumed fraction of susceptibles is 20% for *Random* and 10% for the other five mechanisms.

For all six mechanisms, the current decrease in new case detection of leprosy is predicted to continue over the next decades (Figure 4). The decrease is slowest for both genetic mechanisms and fastest for *Household* and *Random*. The mechanisms that combine *Household* and the genetic mechanisms take an intermediate position.

## Discussion

Different mechanisms for heterogeneity of leprosy susceptibility can explain the observed clustering in household contacts of leprosy patients. The fit to aspects of the data – new case detection rate, household size, or relationship – depends on the assumed mechanism for heterogeneity in leprosy susceptibility. The predicted future decline in the new case detection rate also depends on these mechanisms. For this study, we had access to a large and detailed data set on clustering of leprosy within households,[34] and data from the same region providing essential information about household composition.[26] We used these data to quantify our microsimulation model. However, even with this large and detailed data set, we could not determine the most likely mechanism responsible for the heterogeneity of leprosy susceptibility, or even exclude one of the hypothesized mechanisms.

The most comprehensive dataset available on the contacts of leprosy patients was used in our study and even with this data and a very elaborate model it was not possible to distinguish between different mechanisms of leprosy susceptibility. One reason could be, that even in this large dataset among contacts only 43 new previously undiagnosed patients were present. Furthermore, the household factor is a great unknown in our model. This factor can be a very complicated and maybe time dependent, such as episodes of food shortage and malnutrition. Our model results indicate that in-depth studies (e.g. into the role of starvation and malnutrition) or large-scale human genetic and heredity studies could elicit more precisely the nature of the heterogeneity in susceptibility for leprosy.

Our model only takes into account ‘within-household’ and ‘population level’ transmission. Especially the population level contact structure is simplified extensively. Population contacts are modeled in such a way that every individual has the same chance to encounter every other individual in the population. Of course in reality due to social structures and physical boundaries the contacts will focus on smaller groups. The effect on the epidemiology of leprosy might be that certain households have a higher probability of coming into contact with an infected household. However, because of the low prevalence of infection, the contribution to the clustering of leprosy in households, which was the objective of this study, is very limited. Incorporating such social structures is a challenge.

Although the awareness campaigns in Northwest Bangladesh have lifted much to the stigma surrounding leprosy, contact behavior of symptomatic patients might change. It would be expected that contact rates are smaller. This could be modeled in the given framework by introducing a reduction in transmission probabilities from the moment of becoming symptomatic. Nevertheless, the outcomes are not likely to change much, because the majority of infections occur in the asymptomatic phase.

Our model shows that not assuming an explicit mechanism for susceptibility (*Random*) requires that both transmission parameters are much higher than for the other mechanisms. A short time until infection within household, *i.e.* a high *c_hh_*, means that most susceptible household contacts will be infected relatively quickly after becoming a household contact. Without a mechanism that clusters susceptibility in a household, a high probability of infecting susceptible household contacts is needed to obtain the appropriate number of household contacts with leprosy. For the *Random* mechanism, *c_pop_* is substantially higher than for the other mechanisms, which is in concordance with the existing theory that clustering of susceptible individuals in households increases the endemic level of infectious diseases with an equal transmission rate.[35]

Differences in the more detailed model output (Figure 3) give additional insight into the behavior of the assumed mechanisms for heterogeneity of leprosy susceptibility. The six mechanisms differ in the distribution of cases over the household sizes. For the *Household* scenarios, relatively more cases occur in these small households (Figure 3B), as an individual may become susceptible when moving from a non-susceptible household to a susceptible household. Newly created households are small, and usually consist of a recently married couple. The move to a susceptible household after marriage is also reflected in the prevalence among spouses (Figure 3C).

In contrast, the disease prevalence peaks in moderately large households for the genetic mechanisms (Figure 3B). In these genetic mechanisms, the probability of having (related) susceptible housemates in small houses is small, while for larger households many inhabitants are related (siblings, children etc.), and the probability of susceptible housemates is high. Genetic mechanisms tend to underestimate the observed prevalence among spouses. This might be explained by marriage within the extended family, which occurs frequently in many cultures, but which has not been included in our model. The fit to the prevalence among spouses improves when assuming a mix of genetic and household factors responsible for leprosy susceptibility (*i.e. Household* & *Dominant* or *Household* & *Recessive*). Combining the scenarios produces results between the *Household* and genetic mechanism outcomes, with overall good fit, perhaps suggesting that multiple factors determine susceptibility to leprosy. We have fixed the contributions of each mechanism to 50% *Household* and 50% genetic[5], but with even better and more detailed data it may be possible to estimate these contributions more precisely in the future.

In the study area[36], the new case detection is declining, which is likely to continue in the coming years according to our predictions (Figure 4). However, the speed of decline will depend on the assumed heterogeneity mechanism. The speed of decline is observable in the coming decade, and will thus provide a clue to the underlying mechanism. Somewhat surprisingly, the model predicts the fastest decline for both the *Random* and *Household* mechanisms. These mechanisms differ considerably; the *Random* susceptibility is not clustered in households by an explicit mechanism, while the *Household* mechanism strongly clusters susceptibility. These equally fast declines are explained by our choice of 20% susceptibles in the population for *Random*, whereas we chose 10% for the other mechanisms. The difference in speed of decline between the *Household* mechanism and the genetic mechanisms can be explained by the consequences for contact tracing, which, together with self-reporting, is the only way to detect leprosy in the model. For example, while the genetic mechanisms have a higher prevalence among siblings than the non-genetic mechanisms, these siblings will marry other people and form a household of their own, possibly escaping detection. Furthermore, the *Household* mechanism predicts a high prevalence among spouses, who are likely to be picked up by contact tracing.

In conclusion, in this study we have demonstrated that analysis and modeling of a large and detailed data set on contacts of leprosy patients could not unequivocally reveal the mechanism for the heterogeneity in leprosy susceptibility that is responsible for the clustering of the disease in households.

## Supporting Information

File S1 SIMCOLEP, model description and parameterization. Full description of the SIMCOLEP microsimulation model and parameterization.
(2.49 MB PDF)
