# Supplementary material for: Different Mechanisms for Heterogeneity in Leprosy Susceptibility Can Explain Disease Clustering within Households
Source: PLoS One. 2010 Nov 19;5(11):e14061. doi: 10.1371/journal.pone.0014061 (PMC2988824; doi:10.1371/journal.pone.0014061)
Supplement: File S1 — SIMCOLEP, model description and parameterization. Full description of the SIMCOLEP microsimulation model and parameterization. (2.49 MB PDF) [file pone.0014061.s001.pdf]

Description of SIM*CO*LEP

Supplementary information to

"Different mechanisms for heterogeneity in leprosy susceptibility can explain disease clustering within households"

E.A.J. Fischer[$], S.J. De Vlas, A. Meima[#], J.D.F. Habbema, J.H. Richardus*

Department of Public Health, Erasmus MC, University Medical Center Rotterdam, P.O. Box 2040, 3000 CA Rotterdam, The Netherlands *E-mail correspondence: j.richardus@erasmusmc.nl

[$]Current address: Central Veterinary Institute-WUR, P.O. Box 65, 8200 AB, Lelystad, The Netherlands, egil.fischer@wur.nl

[#]Current address: GGD Rotterdam-Rijnmond, P.O. Box 70032, 3000 LP, Rotterdam, The Netherlands.

Background

Leprosy is a disease caused by infection with the bacterium *Mycobacterium leprae*. Leprosy evolves in a spectrum between two poles (tuberculoid and lepromatous leprosy). The infection is eventually cleared for those with tuberculoid leprosy, while the lepromatous form is chronic. Not all people are susceptible to leprosy, and a marked heterogeneity exists in this susceptibility. This may be because of a resistance against infection or a sufficiently fast clearance of the infection to prevent disease[1]. For those developing leprosy, the incubation period of the disease is long, with 4 to 11 years depending on the type of leprosy [2]. Especially contacts of known leprosy patients are at risk of developing leprosy. These individuals have a higher exposure, but could also be more likely to be susceptible than people in the general population due to shared environment –household– or familial relationship to the patient.

Modeling can aid to extrapolate trial outcomes of one study to whole populations or from a short time frame to longer time periods. This provides a way to compare different control strategies. Modeling is also a way of getting insight into underlying natural mechanisms, *e.g.* the aforementioned heterogeneity in susceptibility. Models for leprosy have up to now not taken into account the household structure of a population, and explicit genetic mechanisms[3,4]. We aim to assess dynamics on a household level, thus the household structure of population needs to be incorporated. As leprosy has a long incubation period[1] the timescale at which the disease evolves and the timescale at which households changes are comparable. This means that a household often does not have the same composition at the end of the infection as it was at the moment of infection. Individuals including those infected, can have moved out or new (possibly infected) individuals can have moved into the household. We take up the challenge to explicitly model the formation and change of household [5]. The model will be parameterized for northwest Bangladesh[6] and fitted to the detailed disease data of a trial in the same area[7].

Microsimulation

SIMCOLEP simulates leprosy transmission in a population structured by households that form and dissolve during the simulation. The model is a microsimulation or a stochastic individual-based model [8,9]. The model simulates the life history of fictitious individuals, including the household formation, and the natural history of infection with *M. leprae*. The state of an individual changes during events that are scheduled in continuous time. The timing of events is determined by probability distributions, which is determined by the current state and history of an individual. The model is divided into two modules: a population module, and a disease module. The population module describes processes unrelated to disease or

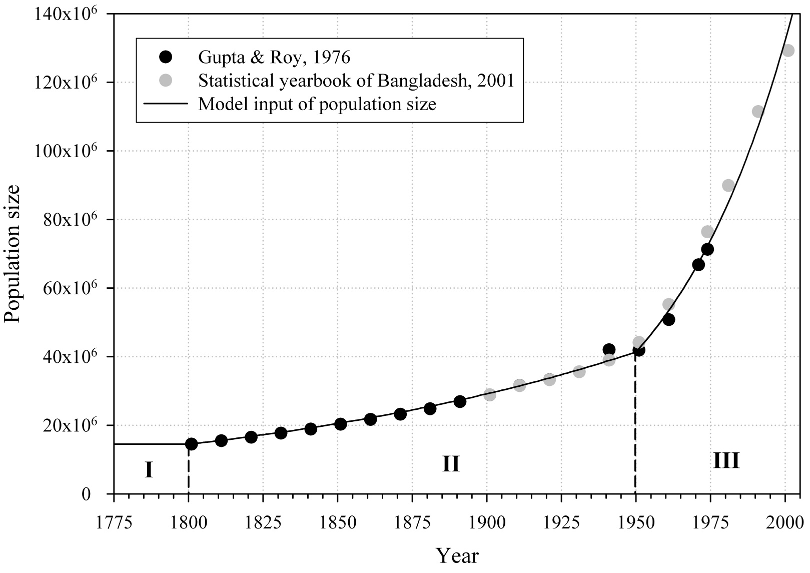

Figure S-1 Population size of Bangladesh from 1775 to 2000. First official census was conducted in 1901; other data points are estimates [10,11]. The solid line is exponential growth curve used as input for the model with three phases: (I) A constant population size (II) slow growth with rate 0.007 y^{-1}, and (III) fast growth with 0.0235 y^{-1}.

infection, such as birth, death and marriage. The disease module simulates processes of infection and disease, including interventions.

A computer program was written in JAVA 1.5.0 [12] to make the calculations of the model using a similar structure as STDSIM [13]. To explicitly simulate an infectious disease requires the simulation of many interacting individuals, and can become computationally demanding. Reliable simulation of a relatively rare infectious disease, such as leprosy, requires a large population. To keep computation time within reasonable limits, we used the MUSIDH method [14] with a setting of 50 disease histories to 1 life history. In short this method implies that every demographic life history (birth, death etc.) is used as if 50 individual have exactly the same demographic life history, while disease events differ between these 50 individuals. This prevents the simulation of many demographic life histories.

Population module

The population grows with a time-dependent growth rate. In total we recognized three population growth phases (see Figure S-1) and choose to model population growth with exponential growth during these three phases. For the population before 1800, we assumed a constant population (*i.e.* a growth rate of 0 y^{-1}). The second phase of slow growth from 1800 until 1950 occurred with a rate of 0.007 y^{-1}. From 1950 onwards the population grows with a rate of 0.0235 y^{-1}. The population growth-curve after 1800 was obtained from extrapolations based on census data [10,15]. The population size is kept at the required size by replacing deaths by births, and population growth is accomplished by additional births. We assumed a closed

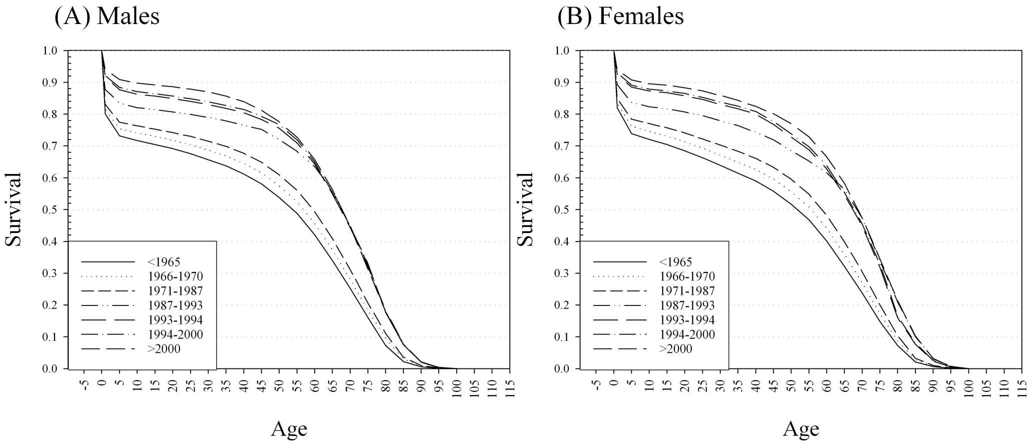

Figure S-2 Input survival curves for males (A) and females (B) in Bangladesh for the years 1961 until 2000 [11]

population, hence no migration. The population size in the simulations was 20,000 in 1800, growing to 195,500 in 2003.

At birth, a new individual is created and the age of death is determined by a sex-dependent survival curve, which changes with calendar time (see Figure S-2). We used the available survival data from 1961 until 2000 [10,15]. Survival data previous to 1961 were not available, and therefore we used the survival curve of 1961 for all years previous to that.

The newly created individual is placed into a household in which a married female is available as mother. The actual mother is randomly selected from all married females weighed by her age. The age-weighed selection of a mother is based upon age specific birth rates [15]. The birth rates for 1995 are shown in Figure S-3.

Unmarried males and females can be coupled during wedding events, which are scheduled such that the proportion of married people in each age group matches census data (Figure S-4 [15]). After the death of a married person, the surviving spouse is again a candidate for marriage again. At marriage, 25% of couples create a new household; for the other couples the female will become member of the household of the male. In the latter case, the household will split up with a rate of 0.083 y^{-1} (*i.e.* after 12 years), and the married couple and their possible children will create a new household.

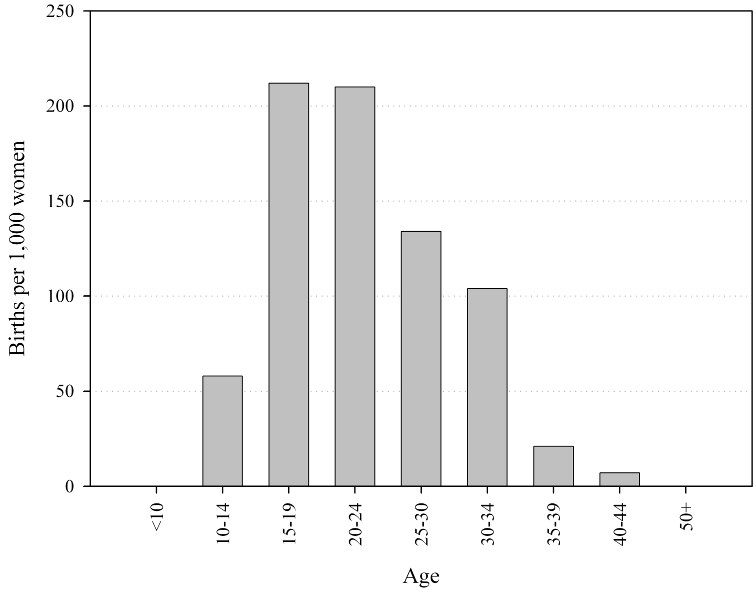

Figure S-3 Example of the input of age specific birth rates for women (Bangladesh, 1995), given as the number of children born to 1,000 women in a specific age group [11].

Movement other than by marriage takes place between households by 30% of non-married males. The age is of movement is chosen randomly from a uniform distribution between 12 and 22 years of age. Twenty percent of these moving males create a new household. For the others a new household is randomly chosen weighed by the size of the household. The weight is 0.25 for households of size 1 and increase linearly to 1.0 at 4 and then linearly decreases to a weight of 0.0 for households of size 50. Hence, movement to households of size 4 is most likely, that become households of size 5 after movement. In the simulations, household sizes maximized at 25 inhabitants.

Data to directly quantify the parameters for the model of movement of people are unavailable, and therefore the above-mentioned values were obtained by calibrating the model to mimic the distribution of household sizes in Nilphamari district in Bangladesh and the percentage of people that moved during a 2-year period. The observed average household size was 4.6 (ranging from 3.9 to 5.9 between villages), and 3.1% of the population moved per year (ranging from 2.0% to 3.6% between villages) [6]. The calibration of parameters gave an average household size of 4.3 and the movement rate was 2.9% per year. Simulated household sizes were slightly larger than observed, but the household size distribution did not differ significantly from the data [6] (χ^2 test, p = 0.25, Figure S-5).

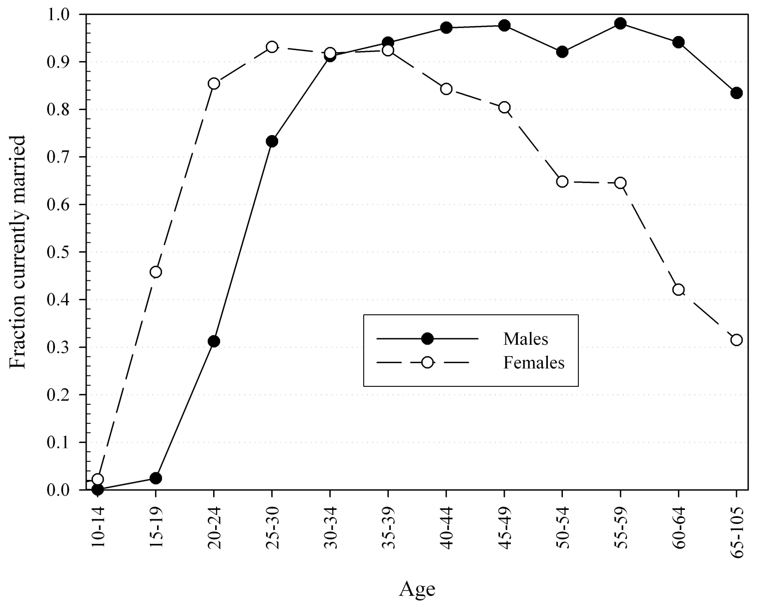

Figure S-4 Example of the input of fraction males and females per age group currently married (Bangladesh,1991) [11].

Disease module

The disease module exists of four separate, but interacting components: transmission, natural history of infection, allocation of susceptibility and type of leprosy, and interventions.

Transmission

Transmission occurs during events in which an infectious individual has contact with a susceptible individual. We modeled two transmission processes (1) in the general population and (2) an additional within-household transmission. The contacts in the general population are made indiscriminately to people within and outside the household of the infectious individual, while the within-household transmission takes place during contacts of household members.

With a contact between two individuals is meant that this contact event is “close-enough for transmission” of the infection. The actual probability of transmission during these close-enough contacts is scaled by the infectivity function. The infectivity function, $A(\tau)$, is the probability of transmission as a function of the time since infection, τ . Here, the infectivity function is a continuous linear function from 0 to 1 during the asymptomatic state, and constant at 1 during the symptomatic state. Transmission events from an infectious individual to other individuals in the general population are timed according to a non-stationary Poisson process [16] with the rate function determined by the product of the population contact rate, c_{pop},

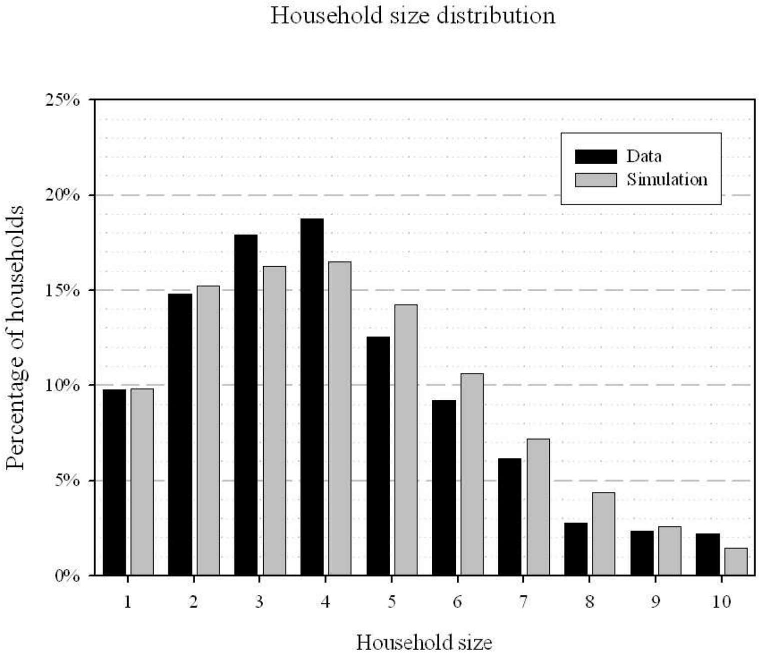

Figure S-5 Result of calibration of simulation population module to the observed distribution of household size in Northwest Bangladesh in 2006(N = 859)[6]. There is no significant difference between data and simulated distribution (p = 0.25, χ^2-test).

and the infectivity function, *A(t)*. Equation S-1 gives the expected number of events during the period 0 to *t*. The next event is found by determining for a random variate *U* the expected time until 1 transmission event making use of the inverse of Equation S-1, Λ_{pop}^{-1} [16]. Such a transmission process is called frequency dependent transmission (or mass action)[17], which means that the number of contact events per individual per time unit (*i.e.* year) is independent of the population size.

$$\Lambda_{pop}(t) = c_{pop} \cdot \int_0^t A(\tau)d\tau \qquad \text{Eq. S-1}$$

Additional to these infections, a within-household transmission process is modeled. A susceptible living in a household with one or more infectious individuals can be infected within the household. The within-household transmission process is modeled by density dependent transmission (or pseudo mass action)[17], which means that the number of contact events per individual per time unit increases with the household size. The rate at which susceptible individuals are infected is determined by all infectious individuals in a household and the within-household contact rate, c_{hh}. For each couple of an infectious individual and a susceptible individual in a household, a transmission event is determined by c_{hh} and *A(t)* similar to Equation S-1. The susceptible individual will be infected during the first

transmission. With I infectious individuals in a household the time until the transmission event is determined for I random variates of a uniform distribution ($U_{1, \ldots,} U_I$) producing the minimal time until the transmission event.

$$T_{transmission} = \min\{\Lambda_{hh}^{-1}(-\ln U_1),\ldots,\Lambda_{hh}^{-1}(-\ln U_I)\} \qquad \text{Eq. S-2}$$

Natural history of infection

The value of the infectivity function, the probability of detection and the rate of self-reporting depend on the state of the infection. The infection is modeled by discrete infection states. After infection individuals are either in the asymptomatic state, the symptomatic state, or the recovered state. We used the structure and estimates for the natural history of the infection as Meima *et al.* [2].

Infection and disease variables, such as detection probability and the infectivity function, have a value corresponding to the infection state, or for the infectivity function the proportion of time spent in the state. The model distinguishes never-susceptible and susceptible individuals. Simulations are done for 5%, 10% or 20% susceptibles in the population. Of the susceptible individuals a fraction of 80% will go through a self-healing infection, and the remaining 20% of susceptibles becomes chronically infected [1,2,18].

The self-healing type is never infectious [2]. The duration of the asymptomatic state is gamma distributed with mean 4.2 years and a standard deviation of 1.9 years[1,2]. In the symptomatic state, the self-healing type is detectable during examination and will be treated immediately after infection. The self-healing type is uninfected, and recovered without symptoms at the moment of self-healing. The time until self-healing from onset of symptoms is exponentially distributed with rate 0.2 (*i.e.* mean duration of 5 years). The self-healing type is assumed never to be infectious.

The chronic infection has an asymptomatic period with mean 11.1 years and standard deviation of 5.0 years, and will be symptomatic until treatment or death of the individual. During the asymptomatic period the infectivity of an individual, *i.e.* the probability of infecting during a sufficiently close enough contact, increases linear to one at first symptoms.

Treatment is given directly at detection and makes an infectious individual immediately non-infectious. Relapse of disease after treatment for both chronic and self-healing infections occurs with a rate depending on calendar time. Between 1970 and 1990 dapsone monotherapy is given, and relapses occur with a rate of 0.015 y^{-1}, and after full implementation of multi-drug therapy (MDT) in 1990 the relapse rate is 0.001 y^{-1} [19]. Of all treated cases including those of the self-healing type, 90% will relapse as a chronic infection, and 10% as a self-healing infection[20].

Allocation of susceptibility and type of leprosy

The susceptibility of an individual is determined by one of six mechanisms of allocation of susceptibility and of the type of leprosy (self-healing or chronic infection):

- **Random**, Equal probability for each individual, *i.e.* random allocation of susceptibility and type of leprosy
- **Household**; Random sample of individuals in randomly selected households
- **Dominant** genes inherited from one or both parents; both susceptibility and the type of leprosy
- **Recessive** genes inherited from both parents; both susceptibility and the type of leprosy
- 50 % by **Household** and 50% by **dominant** genes
- 50 % by **Household** and 50% by **recessive** genes

For **Random**, individuals are determined to be never susceptible, self-healing or chronic randomly at birth. For **Household**, when a household is created, it is determined whether it contains susceptible inhabitants with in total 25% of the households containing susceptibles[21]. However, not all inhabitants of such a household will be susceptible, and at birth, it is determined whether or not an individual is susceptible, when living in a susceptible household. For the three percentages of susceptibility in the population, 5%, 10% and 20%, respectively 20%, 40% and 80% of the inhabitants of the household is susceptible. The type of leprosy (self healing or chronic) is determined randomly for susceptible individuals. The genetic mechanisms are governed by two[22] genes (one for susceptibility and one for the type of leprosy). These genes are both either **dominant** or **recessive**. Children inherit one allele of a gene from both parents. The final combination of alleles – the genotype – then determines the phenotype consisting of susceptibility and type of leprosy. The fifth and sixth mechanisms are combinations of **Household and dominant** and **Household and recessive**. In these mechanisms, half of the susceptibles was susceptible due to their genetic make up, and the other half due to living in a susceptible household [23].

Due to the length of simulations (over 1000 simulated years) genetic drift causes divergence from the starting frequencies of phenotypes in the genetic scenarios. The proportion of alleles at the start of the simulations is taken such that the percentage susceptibles is 5%, 10% or 20% during the last 50 years of the simulations.

Leprosy control

The leprosy control program starts in 1970 with passive case detection and treatment. Detection delays are gamma distributed, and start with mean 12 years and standard deviation 3.5 years in 1970, and decreases to a mean of 2 years (standard deviation 1.4 years) in 1994 [24]. If a self-healing infection heals before the randomly determined passive case detection, the 'case' will not be detected. At the moment of passive case detection, the individual is

diagnosed based on the infection state. Two diagnoses are possible mild disease and severe disease. Mild disease is the diagnosis for the symptomatic state of the self-healing type and severe disease for the symptomatic state of the chronic type.

Household members of a detected case are subject to contact tracing (*i.e.* active case detection) from 1990 onwards. During contact tracing, the probability of a positive diagnosis is determined by the detection probability of the infection state of an individual. Contacts are followed up yearly for 3 consecutive annual visits at each of them 90% will be examined. Contacts can be diagnosed as “no disease” for individuals that are uninfected and in the asymptomatic states; furthermore 10% symptomatic cases are missed during examination and thus incorrectly given the diagnosis “no disease”. The remaining 90% of symptomatic cases is diagnosed as mild disease for self-healing infections or severe disease for chronic infections.

BCG, a vaccine used against tuberculosis, has a protective effect against leprosy. In this study, we choose a life-long protective effect of 60% against infection with *M. leprae* [25,26]. Only BCG vaccination prior to infection with *M. leprae* has a protective effect. BCG vaccination of newly born children starts in 1974. The model starts with a BCG campaign in 1974 in which 40% of all children between age 0 and 10 are vaccinated. From 1975 until 1980, 40% of children are vaccinated. From 1980 until 1990 the BCG vaccination coverage increases up to 80% and on that level it remains until the end of simulations[26,27].

Estimation of contact rate parameters, c_{pop} and c_{hh}

The two contact rates, c_{pop} and c_{hh}, were estimated by fitting the model to data from the DBLM registers in Nilphamari, and a study among contacts of leprosy patients by Moet *et al*[7]. The model was fitted to three aspects of the data: (1) new case detection in 2003, (2) prevalence among contacts by 6 household size categories, and (3) the distribution of previously undetected cases among household contacts for 5 categories of relationship to the index patient. The microsimulation model produces estimates for each aspect of the data set under different values of c_{pop} and c_{hh}, which are compared to data by a log-likelihood function.

The microsimulation model produces a new case detection rate as a function of c_{pop} and c_{hh}, denoted by $\lambda(c_{pop}, c_{hh})$. As matter of convenience, we will drop the notation for simulation outcomes as a function of the contact rates (*e.g.* λ means $\lambda(c_{pop}, c_{hh})$). In this section we reserve Greek letters for the simulation outcomes and a Latin letter for data. The log-likelihood of the observed number of new case detection k is determined assuming a Poisson distribution with simulation outcome rate λ (Equation S-3).

$$L_{NCDR}(\lambda \mid k) = \ln\frac{1}{k!} + k\ln(\lambda) - \lambda \qquad \text{Eq. S-3}$$

The second aspect of the dataset determines the fit to the prevalence of previously undetected cases among household contacts. The parameters, α_i, are the simulated rates of the Poisson distribution for the size household size categories: 2+3, 4, 5, 6, 7, 8 or more inhabitants. Variables s_i indicate the observed number of cases for household size category i. The log-likelihood is the sum of Poisson log-likelihoods for all household sizes (Equation S-4).

$$L_{hhsize}(\alpha_1....\alpha_6 \mid s_1...s_6) = \sum_{i=1}^{6}\left(\frac{1}{s_i!} + s_i\ \ln(\alpha_i) - \alpha_i\right) \qquad \text{Eq. S-4}$$

The third aspect determines the fit to the data on 5 categories of relationships to the index patient: spouse, child, parent, sibling or other relationships. The simulated probability for a person of relationship category i of being cases is indicated by π_i. The variables, r_i, give the number of cases in a relation category i. The total number of contacts of a certain relationship category is indicated with $n_{i.}$. The log-likelihood is the sum of Binomial log-likelihoods for all relationships (Equation S-5).

$$L_{relation}(\pi_1...\pi_5 \mid r_1...r_5, n_1...n_5) = \sum_{i=1}^{5}\left(\frac{n_i!}{r_i!(n_i - r_i)!} + r_i\ln(\pi_i) + (n_i - r_i)\ln(1-\pi_i)\right) \qquad \text{Eq. S-5}$$

The overall log-likelihood was used to determine the fit of the combination of contact rates. The combination of contact rates with the highest log-likelihood is the best model quantification. The fit to all datasets are combined in the log-likelihood function S-6. Constant *C* is the sum of the parts of Equations S-3 to S-5 that do not depend on simulation outcomes, only on the data, and are therefore equal for any assumed combination of c_{pop} and c_{hh}. This constant can be ignored for the maximization.

$$\begin{aligned} L(\lambda,\alpha_1...\alpha_6,\pi_1...\pi_5 \mid k,s_1...s_{6,},r_1...r_5,n_1...n_5) = C + \\ + k\ln(\lambda) - \lambda \\ + \sum_{i=1}^{6}\left(s_i \ln(\alpha_i) - \alpha_i\right) \\ + \sum_{i=1}^{5}\left(r_i \ln(\pi_i) + (n_i - r_i)\ln(1-\pi_i)\right) \end{aligned} \qquad \text{Equation S-6}$$

$$C = \ln\frac{1}{k!} + \sum_{i=1}^{6}\frac{1}{s_i!} + \sum_{i=1}^{5}\frac{n_i!}{r_i!(n_i - r_i)!}$$

The log-likelihood ratio is the difference between the values of Equation S-6 for two different parameters sets obtained from two different models. The log-likelihood ratio times -2 is approximately χ^2-distributed, which can be used to test whether two models are significantly different.

Metamodel

We use a regression model as metamodel [28] fitted to the 11 x 11 parameter grid of simulations (Figure S-6 an S-7). The regression model is derived for the section of the grid in which the minimum is found. The regression model can be used to determine the optimal parameter combination. The likelihood ratios were fitted to a polynomial regression model (see eq. S-7).

$$f(c_{pop}, c_{hh}) = b_0 + b_1 \cdot c_{pop} + b_2 \cdot c_{hh} + b_3 \cdot c_{pop} \cdot c_{hh} + b_4 \cdot c_{pop}{}^2 + b_5 \cdot c_{hh}{}^2 \qquad \text{Eq. S-7}$$

The regression model Equation S-7 was estimated for all possible combinations of linear and log-transformed outcomes and parameters. For each scenario, the regression model with the transformations yielding the highest adjusted R^2 –coefficient of determination– was used as metamodel. The metamodels were used to determine the best fitting parameter combination. The metamodel is used to find the optimal parameter values and the 95% confidence around such an optimum. Figure S-6 and S-7 show the results of the simulation grids, and plots of the metamodels. Thereafter, the log-likelihood was determined by the median of 9 times 100 runs of the simulation model for these parameter combinations (see table S-1).

Table S-1 Estimated best fitting parameter combination calculated from the metamodel. The model fits are determined by the median of 9 times 100 runs.

**Mechanism**	c_{pop}	c_{hh}	**Model fit**			**Total fit**
			L_{NCDR}[a]	$L_{Hh\ size}$[b]	$L_{relationship}$[c]	L
5 % susceptibles						
**Random**	-	-	-[d]	-[d]	-[d]	-[d]
**Household**	11.50	11.07	1.1	2.3	13.1	16.5
**Dominant**	23.10	11.27	0.1	7.9	34.7	42.8
**Recessive**	27.36	8.76	4.1	10.2	38.3	52.6
**Household & dominant**	12.49	8.96	0.0	5.3	16.1	21.4
**Household & recessive**	13.57	13.70	1.4	8.2	17.8	27.5
10% susceptibles						
**Random**	5.70	2.44	0.5	19.3	28.5	48.4
**Household**	2.90	0.22	2.1	6.7	6.6	13.4
**Dominant**	3.35	0.49	1.3	5.8	13.0	20.0
**Recessive**	3.79	0.82	1.1	5.0	23.0	29.1
**Household & dominant**	2.90	0.20	0.5	6.3	7.4	11.4
**Household & recessive**	3.03	0.98	0.0	4.9	8.7	13.7
20% susceptibles						
**Random**	1.33	0.98	0.4	5.0	7.0	12.4
**Household**	1.08	0.05	3.5	6.5	8.9	18.9
**Dominant**	-	-	-[d]	-[d]	-[d]	-[d]
**Recessive**	0.83	0.17	0.0	4.7	17.6	27.5
**Household & dominant**	0.87	0.08	0.1	3.6	10.8	14.5
**Household & recessive**	0.98	0.11	3.5	3.7	9.2	16.4

[a] New Case Detection Rate, Equation S-4 minus constants [b] Household size, Equation S-5 minus constants [c] Relationship with patient, Equation S-6 minus constants [d] Only very poor fits

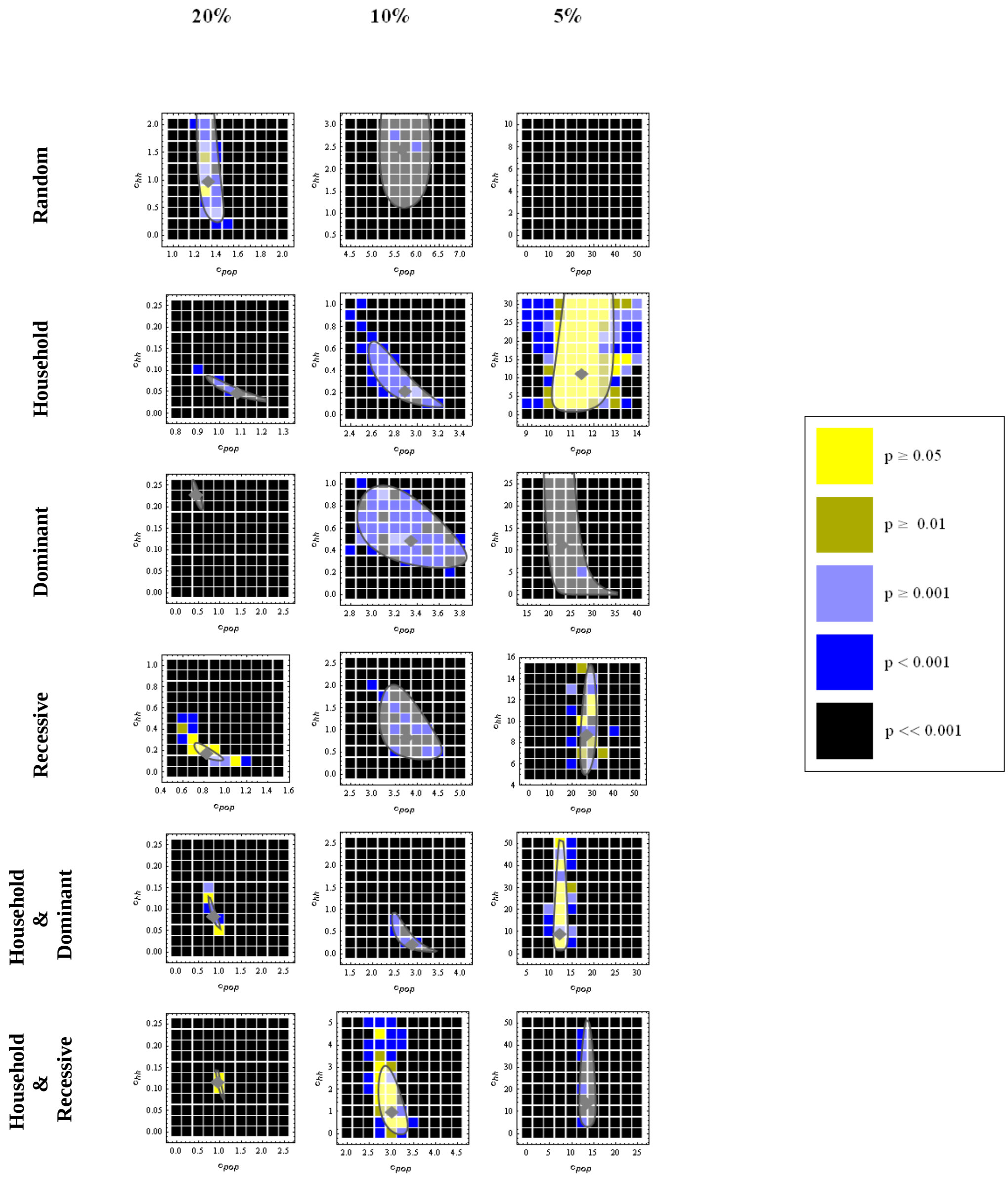

Figure S-6 Fit to data. Simulated values and meta-models for all 18 scenarios. The color of the simulated grid points indicates the difference with the log-likelihood of *the data*. The grey diamond indicates the location of the best fitting parameters values resulting from the minimum of the metamodel. The surrounding thick bordered gray area gives the 95%-confidence area of the parameters. For each panel, the color of the simulated grid points indicates the difference with the minimum of the metamodel (i.e. the value for the parameter values indicated by the gray diamond).

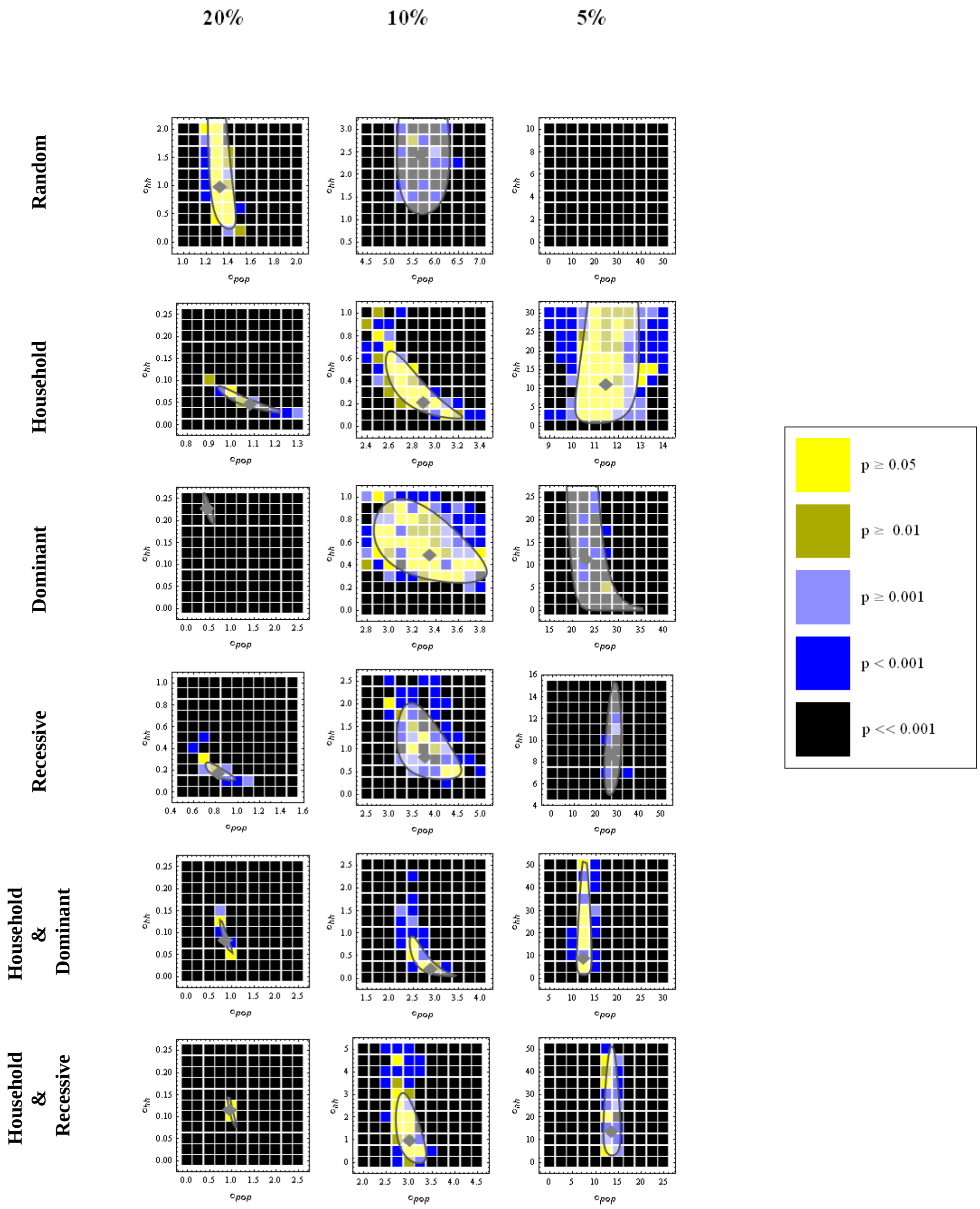

Figure S-7 Fit relative to estimated best fit. Simulated values and meta-models for all scenarios. The color of the simulated grid points indicates the difference with the minimum of *the best of all* metamodels. The gray diamond indicates the location of the best fitting parameters values resulting from the minimum of the metamodel. The surrounding thick bordered gray area gives the 95%-confidence area of the parameters.

REFERENCES

1. Fine PE. Leprosy: the epidemiology of a slow bacterium. *Epidemiological Review* 1982;**4**:161-188.
2. Meima A, Smith WC, van Oortmarssen GJ, Richardus JH, Habbema JD. The future incidence of leprosy: a scenario analysis. *Bulletin of the World Health Organisation* 2004;**82**(5):373-380.
3. Lechat MF. Epidemiometric modelling in leprosy based on Indian data. *Leprosy Review* 1992;**63 Suppl 1**:31s-39s.
4. Meima A, Gupte MD, van Oortmarssen GJ, Habbema JD. SIMLEP: a simulation model for leprosy transmission and control. *International Journal of Leprosy and Other Mycobacterial Diseases* 1999;**67**(3):215-236.
5. Riley S. Large-scale spatial-transmission models of infectious disease. *Science* 2007;**316**(5829):1298-301.
6. Fischer EAJ, Pahan D, Chowdhury SK, Oskam L, Richardus JH. The spatial distribution of leprosy in four villages in Bangladesh: An observational study. *BMC Infectious Diseases* 2008;**8**(125).
7. Moet FJ, Pahan D, Schuring RP, Oskam L, Richardus JH. Physical distance, genetic relationship, age, and leprosy classification are independent risk factors for leprosy in contacts of patients with leprosy. *Journal of Infectious diseases* 2006;**193**(3):346-353.
8. Habbema JD, De Vlas SJ, Plaisier AP, Van Oortmarssen GJ. The microsimulation approach to epidemiologic modeling of helminthic infections, with special reference to schistosomiasis. *American Journal of Tropical Medicin Hygiene* 1996;**55**(5 Suppl):165-9.
9. Smith T, Killeen GF, Maire N, Ross A, Molineaux L, Tediosi F, Hutton G, Utzinger J, Dietz K, Tanner M. Mathematical modeling of the impact of malaria vaccines on the clinical epidemiology and natural history of Plasmodium falciparum malaria: Overview. *American Journal of Tropical Medicin Hygiene* 2006;**75**(2 Suppl):1-10.
10. Gupta AD, Roy SG. Population Estimates for Bangladesh: The Use of a Specific Transitional Population Model. *Population Studies* 1976;**30**(1):15-33.
11. Bangladesh-Bureau-of-Statistics. http://www.bbs.gov.bd/. Vol. 2006. Dhaka, 2006.
12. Anonymous. JAVA. Vol. 2005.
13. Van der Ploeg CPB, Van Vliet C, De Vlas SJ, Ndinya-Achola JO, Fransen L, van Oortmarssen GJ, Habbema JDF. STDSIM: A Microsimulation Model for Decision Support in STD Control. *Interfaces* 1998;**28**(3):84-100.
14. Fischer EAJ, De Vlas SJ, Richardus JH, Habbema JDF. MUSIDH, multiple use of simulated demographic histories, a novel method to reduce computation times in

microsimulation models of infectious diseases. *Computer Methods and Programs in Biomedicine* 2008.

15. Bangladesh-Bureau-of-Statistics. Statistical Yearbook of Bangladesh. Vol. 1. Dhaka: Bangladesh Bureau of Statistics, Statistics division, Ministery of Planning, Government of the People's Republic of Bangladesh, 1995;646.
16. Law AM, Kelton WD. Chapter Eight: Generating Random Variates. *Simulation Modeling and Analysis*. McGraw-Hill series in industrial engineering and management science McGraw-Hill Higher Education; 2000:437-495.
17. Keeling MJ, P. R. Modeling infectious diseases in humans and animals Princeton University Press, 2008.
18. Noordeen SK. The Epidemiology of leprosy. In: Hastings RC, ed. *Leprosy*. Edinburgh: Churchill Livingstone; 1985.
19. Anonymous. Report of the International Leprosy Association Technical Forum. Paris, France, 22-28 February 2002. *International Journal of Leprosy and other Mycobacterial diseases* 2002;**70 (suppl.)**:S1-S62.
20. Smith TC, Richardus JH. Relapse rates in patients treated with dapsone monotherapy and combinations of dapsone and thiambutosine, thiacetazone, isoniazid and streptomycin in the pre-MDT era. *International Journal of Leprosy and Other Mycobacterial Diseases* 1994;**62**(3):353-358.
21. UNSTATS. Millenium Developments Goal Indicators. United Nations Statistical Division, 2000.
22. Mira MT, Alcais A, Van Thuc N, Thai VH, Huong NT, Ba NN, Verner A, Hudson TJ, Abel L, Schurr E. Chromosome 6q25 is linked to susceptibility to leprosy in a Vietnamese population. *Nature Genetics* 2003;**33**(3):412-415.
23. Bakker MI, May L, Hatta M, Kwenang A, Klatser PR, Oskam L, Houwing-Duistermaat JJ. Genetic, household and spatial clustering of leprosy on an island in Indonesia: a population-based study. *BMC Medical Genetics* 2005;**6**:40.
24. Nicholls PG, Croft RP, Richardus JH, Withington SG, Smith WC. Delay in presentation, an indicator for nerve function status at registration and for treatment outcome--the experience of the Bangladesh Acute Nerve Damage Study cohort. *Leprosy Review* 2003;**74**(4):349-56.
25. Setia MS, Steinmaus C, Ho CS, Rutherford GW. The role of BCG in prevention of leprosy: a meta-analysis. *Lancet Infectious Diseases* 2006;**6**(3):162-70.
26. Schuring RP, Richardus JH, Pahan D, Oskam L. Protective effect of the combination BCG vaccination and rifampicin prophylaxis in leprosy prevention. *Vaccine* 2009.
27. Bangladesh reported immunization coverage.

28. Law AM, Kelton WD. Chapter Twelve: Experimental Design, Sensitivity Analysis, and Optimization. *Simulation Modeling and Analysis*. McGraw-Hill series in industrial engineering and management science McGraw-Hill Higher Education; 2000:622-668.
